# Antibacterial Resistance, Wayampis Amerindians, French Guyana

**DOI:** 10.3201/eid1006.031015

**Published:** 2004-06

**Authors:** Karine Grenet, Didier Guillemot, Vincent Jarlier, Brigitte Moreau, Stéphane Dubourdieu, Raymond Ruimy, Laurence Armand-Lefevre, Pierre Bau, Antoine Andremont

**Affiliations:** * Assistance Publique-Hôpitaux de Paris, Paris, France;; † Pharmaco-Épidémiologie Institut Pasteur, Paris, France;; ‡ Groupe Hospitalier Pitié-Salpêtrière, Paris, France;; § Centre Hospitalier de Cayenne, French Guyana

**Keywords:** drug resistance, bacterial, gram-negative bacteria, Latin America

## Abstract

Drug resistance in fecal bacteria was high in Wayampis Amerindians who did not take antibacterial agents and were not hospitalized for 1 year. In the Wayampis Amerindians, an isolated traditional community in French Guyana, antibacterial use was 0.64 treatments per person per year. Hospitalization rate was 6.1% per year. Antibacterial drug–resistant bacteria can spread in persons who are not taking antibacterial agents.

Antibacterial-resistant bacteria can spread in persons not taking antibacterial agents. This resistance results from contaminated food, antibacterial drug exposure, and cross-contamination from humans or animals. Antibacterial resistance is high in developing countries (*1*) because of self-medication, the suboptimal quality of antibacterial drugs, and poor community and patient hygiene (*2*).

To analyze the role of cross-transmission on the resistance of fecal commensal enterobacteria, we conducted a study from October 1 through 15, 2001, among Wayampis Amerindians who lived in the most southern part of French Guyana, in an isolated, ethnically homogeneous, traditional community. The community was made up of 184 males and 204 females (193 children <15 years and 195 adults), who were evenly distributed in three villages (Z, n = 248; TS, n = 85; and YP, n = 55). Access to the villages was restricted to residents, and the sites were isolated, 100 km south of the closest village. Villagers shared large huts (13.9 + 8.6 inhabitants per hut [range 4–38]) with no latrines or hygienic facilities, and used a single spot on the river for drinking, bathing, and disposal of human waste. They ate only local food (crops grown in a traditional manner and meat from fishing or hunting). They did not raise farm animals except a few free-running chickens. A paramedic officer permanently residing in Z provided the only antibacterial agents in the vicinity and recorded their dispensation. When necessary, the villagers were hospitalized in Cayenne, the capital of French Guyana. Medical care was free.

## The Study

Rates of antibacterial exposure were calculated as the ratio of the number of treatments prescribed divided by the number of villagers and compared by using analysis of variance. Chi-square-tests or Fisher exact tests were used for binary variables. During the year preceding the study, 24 (6.1%) of 388 villagers had been hospitalized; 235 (60.6%) had received no treatment with antibacterial agents. One hundred fifty-three (39.4%) of the villagers had received 250 courses of antibacterial treatments. Of the therapeutic agents used, 72 (28.8%) were aminopenicillins, 111 (44.4%) were metronidazole, 36 (14.4%) were macrolides, and 31 (12.4%) were different antibacterial agents (17 penicillin M, 2 penicillin G, 7 cotrimoxazole, 3 cyclines, and 2 first-generation cephalosporins). Ninety-eight (25.3%) villagers had received one couse of treatment, 30 (7.7%) received 2 couses of treatments, 13 (3.4%) received 3 courses of treatments, and 12 (2.1%) received >4 courses of treatments. Overall antibacterial and aminopenicillin exposures were significantly higher in village Z, where the paramedical officer resided (Table 1), and in children.

**Table 1:** Antibacterial exposure in 388 Wayampi Amerindians from three villages in southern French Guyana during the year preceding the study^a^

Antibacterial agents	Frequency of antibacterial exposure in (%)				
	TS (n = 85)	YP (n = 55)	Z (n = 248)	Total (N = 388)	p value^b^
Aminopenicillins	5.9	14.6	23.8	18.6	0.007
Metronidazole	16.5	30.9	32.3	28.6	0.15
Macrolides	8.2	1.8	11.3	9.3	0.2
Other	5.8	7.3	8.9	8.0	0.7
Overall	36.5	54.6	76.2	64.4	0.007

^a^Frequency of antibacterial exposure was calculated by dividing the number of all antibacterial courses during the year preceding the study by the number of persons in each village. ^b^Analysis of variance (ANOVA).

In October 1999, one of the investigators (V.J. or A.A.) asked each adult to participate in the study. Children were recruited with the help of the teachers at school. Exclusion criteria were fever, diarrhea, or acute infection, and a stay outside the study zone, a history of hospitalization (verified by Cayenne’s hospital records), or treatment with an antibacterial agent (verified by records of the paramedical officer) during the preceding year. We chose this period for surveillance because it was the longest period for which information was available. Information was verified by one of the investigators.

The study was approved by the Comité Consultatif de Protection des Personnes se prêtant à des Recherches Biomédicale*s* (CCPPRB) of Cayenne and by the administrative authorities of French Guyana and was authorized by the French Ministry of Health. Before participants were included, the study was presented to the villagers, with the aid of the chief of the village who explained that some of them would be asked to participate. Consent was obtained before participants were included in the study.

Study participants were asked to bring fresh stool samples (unformed samples were excluded) to the paramedic officers. The samples were then mixed into brain-heart infusion broth with 10% glycerol and frozen in liquid nitrogen. Upon harvesting, 25 µL of broth was added to 2 mL of brain-heart infusion broth, incubated for 4 h, and plated on cetrimide, Chapman, and bile-esculin-acid agar containing 10 mg/L of vancomycin (after an enrichment step of 18 h in broth containing 1 mg/L of vancomycin) to detect *Pseudomonas aeruginosa*, *Staphylococcus aureus*, and vancomycin-resistant enterococci, respectively. Antibacterial-resistant gram-negative bacteria were detected by using two separate methods. The first method explored the predominant flora. Drigalski agar plates were plated with the fecal dilutions; after 48 h growth, five colonies were randomly selected, identified, and tested for antibacterial susceptibility as described (http://www.sfm.asso.fr). A participant was defined as colonized in the predominant flora with gram-negative bacteria resistant to a given antibacterial agent when at least one strain resistant to this antibacterial agent was isolated. The second method explored the subdominant flora. Drigalski agar plates were supplemented with either ampicillin (10 mg/L), ceftazidime (2 mg/L), streptomycin (20 mg/L), kanamycin (20 mg/L), chloramphenicol (20 mg/L), tetracycline (10 mg/L), or nalidixic acid (50 mg/L) (*3*), used within 24 h after preparation, and kept at 4°C until used. The plates were plated with the fecal dilutions, incubated for 48 h, and inspected for lactose-positive and lactose-negative colonies. A participant was defined as colonized in the subdominant flora with gram-negative bacteria resistant to a given antibacterial agent when at least one colony grew on agar containing the corresponding antibacterial agent. No further identification was performed; for quality control purposes, some positive plates were randomly selected for confirmation of antibacterial susceptibility of the isolates. Because the number were few and unexpected, colonies that grew on agar containing ceftazidime were all identified by conventional methods or 16S RNA gene sequencing (*4*), when needed; their antibacterial susceptibility was tested; and genes encoding for extended spectrum β-lactamase were characterized, when needed, by polymerase chain reaction and sequencing (*5*). When needed, the clonality of isolates was determined by using pulsed-field gel electrophoresis (PFGE) (*6*).

The fecal flora was thus analyzed in a subgroup of 93 volunteers (39 men and 54 women; 41.2% from village TS, 18.1% from village Z, and 23.6% village YP [p < 0.001]) who met the inclusion criteria cited above, representing 93 (23.9%) of 388 villagers and 93 (39.6%) of 235 who had not received antibacterial agents for 1 year. Carriage of resistant gram-negative bacteria in subdominant flora of these 93 volunteers ranged from >90% for those resistant to ampicillin, streptomycin, and tetracycline to 7% for those resistant to nalidixic acid (Table 2), with no significant difference for sex or age. We found no association between the rate of resistance in the study participants and the number persons who used antibacterial agents or of children in the hut. Fourteen participants (all living in village Z but not in the same hut) were colonized by gram-negative bacteria resistant to ceftazidime, including three *Escherichia coli* strains with a similar pattern by PFGE that produced Bla_TEM-52_ extended spectrum β-lactamase. Nine participants (three, one, and five living in villages TS, YP and Z, respectively, and only two in the same hut) were colonized by strains of *Acinetobacter baumannii* sharing the same susceptibility pattern. Two participants (one in village TS and one in village Z) were colonized by strains of *Ochrobactrum* spp. Neither *S. aureus* or *P. aeruginosa* were isolated. One participant from village Z was colonized by a vancomycin-resistant strain of *Enterococcus gallinarum*.

**Table 2:** Prevalence of carriage of gram-negative bacteria resistant to various antibacterial agents in the predominant and the subdominant fecal flora of the 93 study participants^a^

Antibacterial agent	Predominant flora^b^					Subdominant flora		
	*Escherichia coli* (%)	*Klebsiella* sp. (%)	*Enterobacter* sp. (%)	Other (%)	Any (%)	Lactose positive (%)	Lactose negative (%)	Any (%)
Ampicillin	51 (55)	19 (21)	5 (5)	2 (2)	77 (83)	89 (96)	31 (33)	93 (100)
Ceftazidime	0	0	0	0	0	3^c^ (4)	11^d^ (12)	14 (15)
Nalidixic acid	0	0	0	0	0	7^e^ (8)	0	7 (8)
Pefloxacine	0	0	0	0	0	ND	ND	ND
Streptomycin	42 (45)	2 (2)	0	0	44 (47)	83 (90)	19 (21)	86 (93)
Kanamycin	5 (5)	0	0	0	5 (5)	45 (49)	4 (5)	47 (51)
Tetracycline	59 (63)	14 (15)	0	2 (2)	75 (81)	86 (93)	25 (27)	86 (93)
Cotrimoxazole	27 (29)	0	0	0	27 (29)	ND	ND	ND
Chloramphenicol	22 (24)	0	0	0	22 (24)	48 (52)	27 (29)	60 (67)

^a^Accounting for 23.9% (93 of 388) of the global population of the villages studied and not significantly different from it for male/female ratio or age distribution. ^b^Bacteria with intermediate susceptibility were categorized as resistant. ^c^Extended spectrum β-lactamase *E. coli.* ^d^*Acinetobacter baumanni*: 9 (all with the same susceptibility pattern including susceptibility to ureidopenicillins, third-generation cephalosporins, aminoglycosides, and quinolones) and *Ochrobactrum* spp. (identified by 16s RNA genes sequencing): 2. ^e^*E. coli.*

Resistance rates in the predominant flora were from 95% to tetracycline to 0% to ceftazidime and nalidixic acid, with no significant differences between adults and children, men and women, or villages. Although they were not chosen randomly, approximately 40% of the untreated villagers were included, which suggests that the group was representative of the whole community.

Recently, high resistance rates were also reported in remote populations from Bolivia (*7*) and Nepal (*2*). Here, however, we have provided additional information on exposure to antibacterial drugs and hospitalizations of the study participants. In the villages studied, the global antibacterial exposure (0.64 treatments/person/year) was roughly half that of France (*8*), thus close to the mean rate of European Union countries (*9*), which stresses the indirect impact of antibacterial use on persons who do not use them. Unrecorded antibacterial drug use in the participants was unlikely because no alternate sources of antibacterial agents existed, and free antibacterial agents were provided when needed, decreasing the likelihood of nonprescribed use or sharing of antibacterial agents by family members.

Our results can be compared only to those of studies performed with similar methods and in persons who also had not taken antibacterial drugs. For instance, resistance rates of predominant *E. coli* in the Wayampis population to ampicillin, tetracycline, and streptomycin were higher than that observed in French bank workers (*10*) or Bostonian children (*1*), but close to that reported in children from Venezuela and China (*1*). That the resistance rate was higher in the Wayampis Amerindians than in the French workers was unexpected, considering the higher levels of antibacterial drug use in the French community. These data suggested frequent cross-transmission likely attributable to poor hygiene (*1**,**2*). Cross-transmission is common among households, even in industrialized countries (*11*). Its role in the spread of drug-resistant bacteria among the Wayampis Amerindians, even in adults, was further indicated by the lack of difference in resistance rates according to age.

Resistance to quinolones, which were not used in this community, was lower than in Europe (*12*), which illustrates the specificity of selective pressure. Third-generation cephalosporins were also not used. Thus, how samples from three participants were colonized by an *E. coli*–carrying Bla_TEM-52_ (*13*), an extended-spectrum β-lactamase-gene found only in hospitals, is difficult to explain. However, enterobacteria that produced an extended spectrum β-lactamase were prevalent at the time in the Cayenne hospital (B.M., unpub. data). Since the three persons with the Bla_TEM-52_ -colonized samples had not been hospitalized during the previous year, possibly this strain, or a different one, carrying Bla_TEM-52_ was acquired and spread in the community by one of the 24 villagers hospitalized during the year preceding the study. Extended-spectrum β-lactamase can disseminate in the community (*14*). We cannot exclude that the three Bla_TEM-52_ carriers had been hospitalized earlier. If they were hospitalized and colonized during hospitalization, this colonization would not have been likely to have persisted for so long; indeed, carriage of resistant nasal staphylococci can last for months, but resistance in intestinal enterobacteria decreases within 10–20 days after selective pressure ends (*15*).

## Conclusions

Antibacterial agents in the food chain are a source of resistance in industrialized countries (*16*). In our study, food was strictly local but may have been the source of the wild-type naturally resistant *A. baumannii* and *Ochrobactrum* spp. strains that we isolated. Environmental species have also been isolated in Amerindians living in nearby (formerly Dutch) Guyana (*17*).

Because data confirmed the lack of direct antibacterial drug exposure in our study participants, the results demonstrate that, once resistance elements are introduced into a population, moderate use of antibacterial drugs in the environment is enough to maintain them in intestinal bacteria when sanitary conditions are poor.
